# Cross-cultural comparison of plant use knowledge in Baitadi and Darchula districts, Nepal Himalaya

**DOI:** 10.1186/s13002-018-0242-7

**Published:** 2018-06-11

**Authors:** Ripu M. Kunwar, Maria Fadiman, Mary Cameron, Rainer W. Bussmann, Khum B. Thapa-Magar, Bhagawat Rimal, Prabhat Sapkota

**Affiliations:** 1 Ethnobotanical Society of Nepal, GPO Box 19225, New Road, Kathmandu, 44600 Nepal; 2 Department of Geosciences, Florida Atlantic University, Boca Raton, USA; 3 Department of Anthropology, Florida Atlantic University, Boca Raton, USA; 4 Ilia State University, Tbilisi, Georgia; 5 Department of Forest and Rangeland Stewardship, Colorado State University, Fort Collins, USA; 6 Institute of Remote Sensing and Digital Earth, Chinese Academy of Sciences, Haidian, Beijing, China; 7 Department of Forest, District Forest Office, Baitadi, Farwest Nepal Nepal

**Keywords:** Medicinal plants, Use reports, Consensus, Transhumance, Intracultural, Nepal Himalaya

## Abstract

**Background:**

This study seeks to better understand the human-nature interface and to measure the variability of plant use knowledge among cultures, through inter- and intracultural analyses. We compared plant collection, use, and management of two culturally distinct groups (Baitadi and Darchula) of the Nepal Himalaya. They inhabit different physiographic regions, yet share the same ecological landscape, environmental resources, and livelihood challenges. We hypothesized that the elderly, native, and traditional healers living in remote and rural places possess more diverse and detailed knowledge of plant use and conservation than young, non-native, and non-healers.

**Methods:**

A total of 106 people were contacted for interviews, and 100 (68 men and 32 women) agreed to share ethnobotanical, demographic, and socioeconomic information. They were asked about the three most important plants for their socioeconomic benefit, culture, primary health care, and livelihood.

**Results:**

The knowledge of plant collection, use, and its transfer was strongly associated with the cultural heritage whereas the ecogeographical condition influences the ways in which plants are collected and used. The divergent knowledge of plant collection, use, and transfer between the participants of Baitadi and Darchula was significantly (*p* < 0.001) attributed to the cultural heritage of the area. The low consensus of plant use (FiC 0–0.87; IASc 0–0.67) between Baitadi and Darchula district could be due to cultural divergence, varied accessibility, physiographic heterogeneity, and biodiversity uniqueness.

**Conclusions:**

Differences in plant use knowledge may help in diversifying the strategies of plant use in accordance with the livelihood, culture, and environment, and therefore, more studies measuring these aspects can further the ecosystem and cultural health of the region.

**Electronic supplementary material:**

The online version of this article (10.1186/s13002-018-0242-7) contains supplementary material, which is available to authorized users.

## Background

Human communities that inhabit remote and rugged ecosystems use diverse livelihood strategies such as utilizing different ethnoecological environments [1, 2] defined by the availability of plants [3], altitudinal gradient and accessibility [4], culture [5, 6], and adaptation [7]. When there is little arable land, indigenous livelihood strategies include animal husbandry, transhumance, seasonal crop production, and collection, use, and trade of medicinal plants [8–10]. However, changes in lifestyle as a result of globalization, increasing population, land-use change, and climate warming affect these livelihood strategies. Socio-acculturation of mountain people and plants jeopardizes the human-biodiversity linkage in the region [11]. The collection and use of plants, hailed for socioeconomic gain, cultural heritage, and drug development [12–15], has now been threatened due to local people’s changing perceptions and their context-specific socioeconomic and cultural transformations [16–18].

The knowledge and practice associated with the collection and uses of plants vary within any culture, because of the abundance and quality of species, geography of the region, origin of the plants, residence of the people, social status, and relationships within the community [9, 19–22]. Cultural factors are sometimes mediated through local classification systems [23], language [24, 25], human cognition, cultural history [26, 27], beliefs, religion [28, 29], taboos, social networks, and access to information [19, 30]. Different subsets of sociocultural factors such as settlement, population, family size, gender, age, ethnicity, education, economy, occupation, and possession also influence knowledge of plant use [3, 7, 31–35]. Studies have demonstrated that ethnobotanical knowledge increases with an individual’s age and length of residence [36]. Thus, cultural variables seem more essential in explaining community knowledge of collection and plant use [24] in addition to the sustainability of plant resources. A continuous outmigration foments a decline in the number of healers and indigenous knowledge holders [37–41], resulting in weakened indigenous knowledge and use systems [42].

Here, we compared the knowledge of plant collection and use of two subculturally distinct groups inhabiting different physiographic regions within the same ecological landscape with access to similar environmental resources. Cross-cultural studies were parsimoniously studied before 1998 [43], but nowadays, they are increasingly being analyzed [25, 28, 44–46]. Intercultural comparison has practical applications because we can address both the consensus and variations of plant use knowledge. In this paper, we carried out a cross-cultural study focusing on different human groups and how their demographic (gender, age), socioeconomic (ethnicity, education, occupation, land and livestock ownership, and food availability), and cultural (length of residence, settlement, language, household size, and livelihood) variables influence the knowledge of plant use. We hypothesized that the elderly, native, and traditional healers living in remote and rural physiographic condition possess more diverse and detailed knowledge of plant use and conservation than young, non-native, and non-healers.

## Methods

### Study area

The Kailash Sacred Landscape (KSL) is a trans-boundary landscape comprised of parts of the southwestern Tibetan Autonomous Region of China and adjacent parts of northern India and northwestern Nepal [47]. At its heart, high upon the Tibetan Plateau lie Mt. Kailash (6638 masl) and two adjacent lakes (Mansarowar), considered a sacred pilgrimage site by over a billion people practicing five religions [11]. The pilgrimage routes to Mt. Kailash and Mansarowar via Urai Pass (Bajhang, Nepal) and Lipulekh (Darchula, Nepal) augment the cultural history of the region [48–54]. The KSL-Nepal occupies 42% of the total KSL, which covers four mountain districts (Darchula, Humla, Baitadi, and Bajhang) in the far-western part of the country. This is one of the most underdeveloped regions of Nepal and faces numerous conservation and development challenges because of the harsh climate, poor accessibility, marginality, and high level of poverty [47, 55]. These cultural and developmental premises are intertwined with historical accounts. Before the Anglo-Nepalese War (Gurkha War) of 1814–1816, the entirety of Kumaon Garhwal to Bairath (Baitadi), Doti was designated as the Katyuri Kingdom under Nepal administration [56], and the corpus of feudal rites was considered a unifying aspect of culture [57]. There are a number of commemorative pillars erected in about 1200 AD in Dehimandu, Baitadi, and adjoining areas memorializing the victorious warriors of the region [58]. The long history of contact of a community with nature infers a tradition and culture that integrate a high number of indigenous medicinal plants for local livelihood [26]. Only about 15–25% of the KSL geographical area is cultivable [11, 59–62], leading to prolonged poverty, which contributes to environmental challenges and calls for sustainable development of the region [15, 63].

**Fig. 1:**
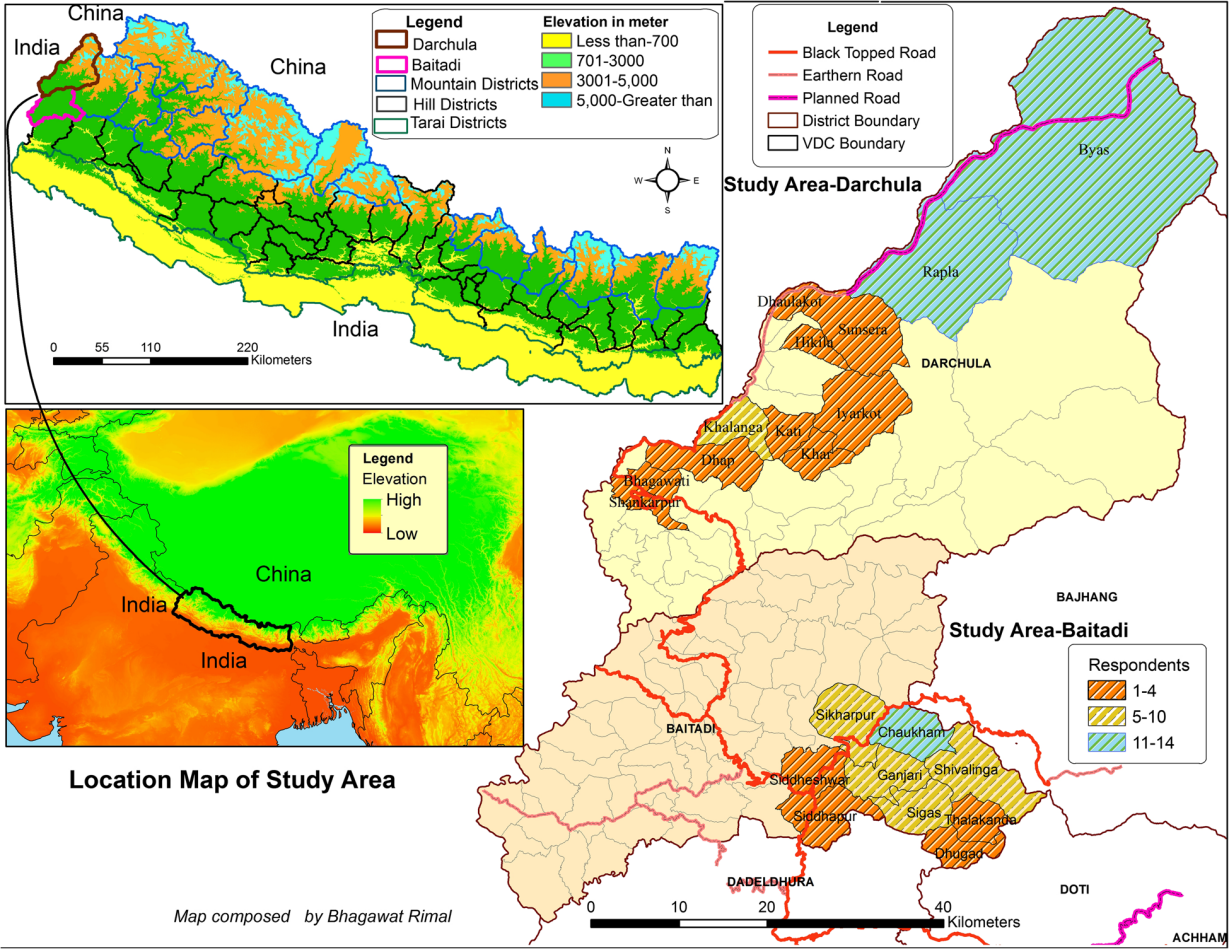
Map showing the location and physiography of the study area and village-wise frequency of respondents

Baitadi and Darchula, the study districts (29° 22′ N to 30° 15′ N/80° 15′ E to 81° 45′ E) located at the westernmost end of the country, represent far-western Nepal bordering India and China (Fig. 1). The study districts Baitadi and Darchula respectively represent hill and mountain ecosystems. Situated in the southern Himalayan foothills, the hill region (Pahad in Nepali) mostly between 700 and 3000 m above sea level (masl) is highly populated, and agriculture is the main form of livelihood [64]. The mountains are regarded as collection grounds for medicinal plants, summer grazing lands, and sacred sites for rituals [65, 66]. Traditional mountain agro-pastoral systems predominate in Darchula and integrate with transhumance, animal husbandry, and medicinal plant collection and trade. These livelihood strategies are adapted for subsistence within the steep terrain and variable climatic conditions. Both study districts, extending up to the southern parts of the KSL, are considered important for growing maize, barley, buckwheat, amaranth, potatoes [67, 68], and relict hemp culture [69].

### Ethnographic setting

There are more than 30 ethnic and 10 minority groups in the study area including the indigenous groups Byanshi and Santhal in Darchula and Kusunda, Dom and Dhanuk in Baitadi [70]. Chhetri is the study area’s dominant ethnic group (about 60%) followed by Brahmin (about 20%), *Dalit* (Lohar/Kami, Sarki, Dhanuk 10%), and others (10%). Chhetri and Brahmin are relatively privileged groups with the highest well-being index [71]. Even though the *Dalit* are receiving reserved access and opportunities provided by the Nepal Government, they are still disadvantaged due to the sociocultural and class system [55, 72]. The sample community of Baitadi District was composed of sedentary farmers and villagers including the hill castes Chhetri, Brahmin, and *Dalit*. Brahmin, Chhetri, and *Dalit* predominate in Baitadi District. Agriculture, wage labor, medicinal plant collection and trade, and traditional healing are the major occupations in Baitadi [35, 73–75]; however, the former contributes the most [10, 76–79]. Baitadi District is renowned for socioculturally designated sacred peaks [80]. Sacred forests are part of the cultural heritage that represent important spiritual sites [81], and local people believe that their livelihood and cultural existence are greatly dependent on the blessings of their deities [65]. The legacy of sacredness, recently demarcated as the Kailash Sacred Landscape, has been shown to have a major effect on culture, conservation, ecology, and environment due to the associated special precautions and restrictions on use [82]. As a result of limited human activity due to sociocultural taboos and prohibitions, sacred places frequently possess old-growth vegetation and many ecologically and socioculturally valuable plant species [83, 84].

The Byanshi is a Tibeto-Burman minority group with a population of about 4000 in the country. About 500 live in Chyanrung and Byansh villages and about 500 in Rapla, Shitola, and Khalanga villages [70]. They are part of a group of people living throughout the Kumaon hills [15], as well as in Darchula (study district), Humla, and Bajhang districts of Nepal [85]. They are semi-nomadic [86], living 6 months in Byansh village (> 3000 masl) in the summer and then descending to the lowlands (Khalanga) for the rest of the year [8]. The Byansh village is covered by snow in winter. They represent the cultural practices and belief systems of the Nepal, farwest, and they speak the Byanshi/Rang language [85], claim to be both Hindu and pre-Buddhist and are well known for their hospitality. Byanshi houses are decorated with fine wood-carved and reddish-brown-painted windows and doors. Like other Buddhists in Nepal, they use *Abies* poles for mounting prayer flags in the yard. *Abies pindrow* Royle (*Himisin*) is a very good resource as prayer flag, fuelwood, furniture, butter churners, medicine, and agricultural implements in mountain communities. Other temperate-alpine medicinal plant species *Angelica archangelica* L. (*Gannanu*), *Dactylorhiza hatagirea* (D.Don) Soo (*Hathajadi*), *Neopicrorhiza scrophulariflora* (Pennell) Hong (*Katuko*), *Ophiocordyceps sinensis* (Berk.) G.H.Sung (*Yartsagumba*), etc. are used and conserved in Darchula District for a long period [87].

The average landholding of a Byanshi family in Darchula District is small and of poor quality, feeding a family only 3 to 4 months in a year [88]. Famines occur often in the district [47, 89] aggravated by limited cultivable land. Collection of hay (grasses) and edible and medicinal plants is a common strategy to offset these hungry periods. In addition, a cash crop, radish is sliced, dried, and taken to the northern border Taklakot and into Tibet to barter for salt. Some high-value medicinal plants are bartered to the lowlands and to India in exchange for food and grains [52]. Widespread collection and bartered species are Yartsagumba (*O. sinensis*), Jimbu (*Allium hypsistum* Stearn), and Satuwa (*Paris polyphylla* Sm.). This trans-boundary trade and transhumance are important livelihood strategies [52, 90].

Forest resources and alpine pastures complement the mountain agricultural system in both study districts, meeting fuel, fodder, timber, and medicinal needs. Fuelwood is commonly used for heating and cooking. Agriculture, livestock, woolen products, and the medicinal plant trade are four major livelihoods maintaining household economies. Because of the remote location, reliance on traditional medicine is associated with wild medicinal plants and on the harmonious existence of spirit and matter. Many traditional healers worship and pray to plants before collecting them, acknowledging the spiritual powers of the vegetation [91, 92]. They believe that plants become more medicinal when processed spiritually and materially [65, 66]. Thus, trade, paired with pastoralism and transhumance in this constrained environment, is a survival strategy [8, 92–94]. The strategic modes of pasture resource utilization are rotational grazing based on a system of transhumance and medicinal plant harvesting [95, 96]. However, the traditional trade and transhumance were disrupted when the trade routes were closed in 1962 because of the Sino-Indian border conflict [97, 98]. This activated contemporary sociocultural and economic transformation, as certain kinds of traditional knowledge began to decline and socio-acculturation and outmigration led people to pursue different economic opportunities to meet new survival challenges [37, 40]. Thus, the traditional subsistence economy in the far-western Nepal Himalaya has experienced a substantial change in recent decades [11, 29, 35, 99] while certain kinds of traditional knowledge in the research area began to decline in recent decades, including that around human-plant relationships.

### Data collection

Informed consent forms were obtained from all oral interview participants written in accordance with the FAU IRB and Nepal research protocols. Inventory-based interviews [100] were carried out with the help of a local assistant and a research associate in each district. Participants were selected based on the dominant groups in the district, elderly people, and occupational affiliations. Only the traditional healers, plant collectors and traders, and elderly people of ages 40–102 were consulted for interviews. Once a traditional healer or a plant collector/trader was identified, snowball sampling was applied to locate and identify peer respondents. The list of traditional healers and plant collectors was referenced from village secretaries, tea vendors, and earlier studies [75, 101].

A total of 106 people were contacted for interviews, and 100 (68 men and 32 women) agreed to share ethnobotanical, demographic, and socioeconomic information. A two-page semi-structured questionnaire was developed in Nepali script prior to the start of fieldwork and administered for interviews. The interviews were carried out during three field visits, the duration of each trip lasting about a month between February and September 2017. A total of 100 participants including 58 from Chhetri, 14 from Brahmin, 24 from Byanshi, and 4 from *Dalit* caste group were interviewed. There were 57 participants including 47 Chhetri, 6 Brahmin, and 4 *Dalit* from 9 villages of Baitadi District and 43 participants including 11 Chhetri, 8 Brahmin, and 24 Byanshi from 12 villages from Darchula district. Informal discussions were held during the evenings while staying with local communities, and sometimes with tea vendors. Tea shops are excellent arenas for observing interactions between communities and discussion of open-ended questions [102].

In the interview, the participants were asked to list the three most important plants for each category, e.g., socioeconomic benefit, culture, primary health care, and livelihood. Demographic information was collected for each participant including socioeconomic status, age, occupation, education, family size, livestock, land ownership, where they migrated from, languages they speak, length of residence, distance of home from district center, nearest health post, and forest. Interviews were supplemented with other investigative techniques, such as participant observation, walk-in-the-woods interviews, and informal meetings [103]. Interviews were conducted individually whenever possible to avoid any direct influences from third parties. The sampling effort was tested by a Jackknife first-order richness estimator 100 permutation species-use curve performed in R. Species-use curve was drawn from the cumulative number of species mentioned as being used versus the number of informants interviewed [104]. While participating in the guided tours, voucher specimens of the species that could not be identified in the field were collected by participants and field assistants and processed and deposited at the Plant Laboratory and Herbarium (KATH), Lalitpur, Nepal, for future reference. Earlier studies carried in and around the study area [60, 75, 98, 100] were used as a taxonomic reference of general species. Plant taxon was verified by using *The Plant List* (Retrieved from www.theplantlist.org).

### Data analysis

Matching information (use reports) from at least three respondents was considered a common response for quantitative analysis [33]. To determine the influence of socioeconomic factors, we used three different indicators of knowledge: (1) use reports, representing the sum of all uses reported by an informant for all species known by that person; (2) useful species, representing the sum of all useful species an informant knew; and (3) use value. Emic use types were later grouped into 19 etic categories for further analyses following Cook [105]. To identify the proportion of culturally important species in each study district, the Index of Agreement on Species (IAS) was calculated following Trotter and Logan [106]:

$$ \left(\mathrm{ns}-\mathrm{nu}\right)/\left(\mathrm{ns}-\mathrm{l}\right), $$

whereby ns is the number of use reports of a given species mentioned by all the participants, and nu is the number of use types attributed to that species. IAS was corrected to Index of Agreement on Species consensus (IASc) for the number of participants who knew a use for the species through the formula:

$$ \mathrm{IASc}=\mathrm{IAS}\times \left(\mathrm{Pu}/\mathrm{Pt}\right) $$

where Pu represents the number of participants who reported a use, and Pt equals the total number of participants interviewed about the species [107]. IASc values vary between 0 and 1, with 0 representing no agreement and 1 total agreement. In this paper, we determined the proportion of plant species with an IASc value > 0.5; this value was chosen as an arbitrary cutoff point for culturally important species following Vandebroek [107].

The frequency of citation of a specific use, that is, the number of individual use reports (nur) for a type of use category, serves to establish the consensus across the respondents [108]. The cultural consensus on a particular use category can help inform efficacy of a plant to that particular use category [106, 109]. The efficacy of plants can be perceived by determining the Fic values. The informant consensus factor (FiC) was calculated as:

$$ \mathrm{nur}-\mathrm{nspp}.\mathrm{used}/\mathrm{nur}-1 $$

where nur shows the number of use reports while nspp shows the number of species used [106]. After analyzing the FiC values of both districts, a comparison was made to sort out the consensus of uses across the two districts. The two main measures of “plant knowledge” consisted of (1) the cumulative number of participants who reported a use for each plant species at the group (cultural) level and (2) the number of plant species used at the level of individual participant. Other measures used to correlate plant knowledge with consensus included the unique use reports (UUR) by a participant.

### Statistical analysis

We grouped the socioeconomic and demographic data into nominal/categorical variable: (1) gender, (2) education, (3) occupation, (4) livelihood type, (5) access to opportunity, (6) food availability, (7) languages spoken and continuous variable: (1) age, (2) household size, (3) livestock size, (4) land size, (5) length of residence, (6) years of healing practice, (7) distance from home to forest, and (8) distance from home to health post. Cross-cultural analysis was made by (1) gender: male and female, (2) education: literate and non-literate, (3) occupation: traditional healers and non-healers, (4) livelihood type: suburban, hill, and sedentary (Baitadi District); rural, mountain and semi-nomadic (Darchula District), (5) food availability: < 6 and > 6 months, (6) language spoken: two languages spoken and more, and (7) access to opportunity: privileged group (Chhetri and Brahmin) and under-privileged (Byanshi and *Dalit*).

Statistical models were used to explore how sociocultural variables interact among themselves and with the knowledge of plant collection, use, and management intensity. We considered *p* values < 0.05 as statistically significant [110]. For count variables, a generalized linear regression model with Poisson logit (link) was used to see the effect of the settlement, length of residence, size of household, livestock and land, and age and experience of participants against plant uses. For categorical variables, emmeans (least square regression of means at logical scale) generalized linear model was used [111]. All the analyses were performed in R studio in R 3.4.1 (R Development Core Team 2017).

## Results

### Useful species and cultural consensus

**Fig. 2:**
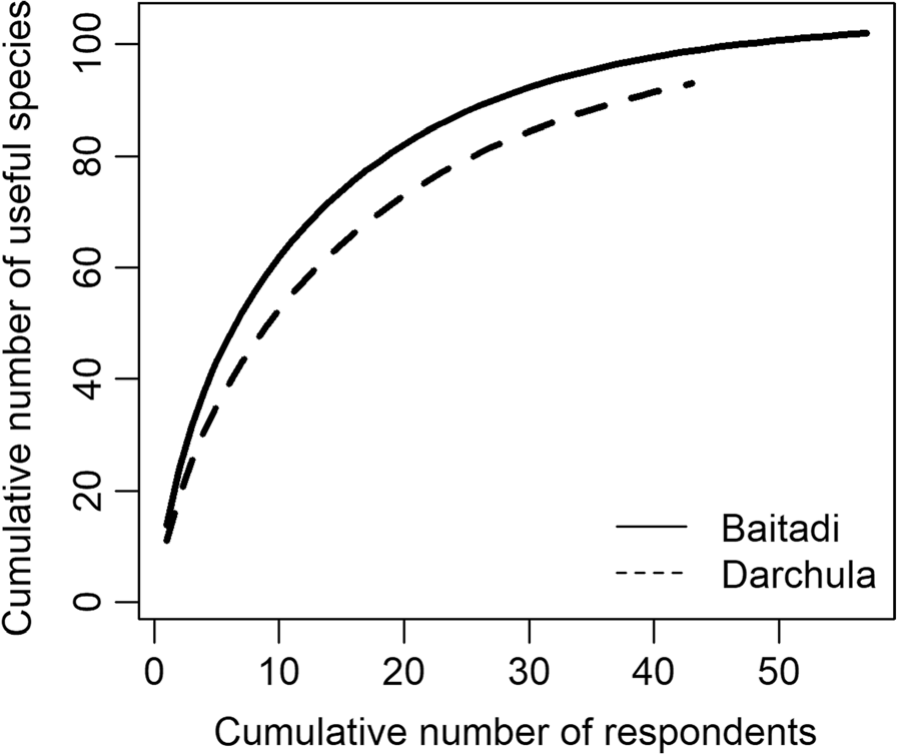
Species-use curve showing the records of the number of useful species against the number of respondents leveled off after 50th participant

A total of 1434 use reports from 122 useful plant species were recorded from 100 participants. Each species was reported for 1–10 use types and 1–63 use reports. The participants from Baitadi District recorded 917 use reports (16.08 person^−1^ use reports) whereas that of Darchula District was 517 (12 person^−1^) (Additional file 1). The use reports were assigned to 89 emic use types later categorized into 19 etic categories for further analyses (Table 1). Of 122 useful plant species, 102 were found useful in Baitadi and 92 in Darchula, with 72 common. The species-use curve approached an asymptote as the number of interviews increased in Baitadi District (57 participants) indicating that there will not be further additions of useful species. However, the curve was not completely leveled-off in Darchula District (43 participants) indicating that further sampling of respondents would yield some new useful species in Darchula District (Fig. 2).

**Table 1:** Emic and etic use categories and informant consensus factor (FiC)

Etic category (abbreviation)	Emic use type	Baitadi			Darchula			Av.
		Use reports	Useful species	FiC	Use reports	Useful species	FiC	
Ritual (Rit)	Ritual, religious, evil spirits, luck	120	18	0.857	85	20	0.773	0.815
Digestive metabolism (Dig)	Diarrhea, dysentery, stomachache, nausea, anthelmintic, appendicitis, gastric, indigestion	102	23	0.782	137	35	0.75	0.766
Infection (Inf)	TB, fever, typhoid, tetanus, leprosy, polio	47	14	0.717	78	19	0.766	0.741
Social materials (Soc)	Wood, fuel, fodder, forage, rope, bedding, agricultural implements	272	36	0.870	28	16	0.444	0.657
Pain inflammation (Pai)	Cuts, wounds, burn, injury, analgesic, toothache, headache	53	23	0.576	45	16	0.659	0.618
Respiratory (Res)	Pneumonia, cold, cough, larynx-sound	58	23	0.614	22	9	0.619	0.616
Livestock (Liv)	Livestock health, veterinary	19	15	0.222				0.111
Musculoskeletal (Mus)	Fracture, sprain, joint pain, backache, bath (rheumatism)	36	16	0.571	24	12	0.521	0.546
Anti-poisoning (Poi)	Snake bite, antidoting, scorpion sting, piscicidal, antileech, insecticidal	21	10	0.55	11	6	0.5	0.525
Food (Foo)	Vegetable, edible, spices	43	20	0.547	10	6	0.444	0.496
Immune (Imm)	Immune, anticancer, nutrition, appetite, growth, tonic	4	3	0.333	18	7	0.649	0.324
Endocrine (End)	Gall bladder, gall stone, diabetes				13	3	0.833	0.416
Genito-urinary (Gen)	Urine infection, hydrocele, piles	9	3	0.75	3	3	0	0.375
Circulatory blood (Cir)	Blood pressure, heart disease, jaundice	15	10	0.357	16	12	0.266	0.311
Household economy (Eco)	Dye, oil, resin	13	6	0.583				0.291
Reproductive (Rep)	Lactation, fertility, conceive, abortion, dudhelo (mammary gland complication)	7	4	0.5	4	4	0	0.25
Skin (Ski)	Acne, scabies, skin swell, hair fall, makada, pilo, pitka (skin rashes)	35	18	0.5	6	6	0	0.25
Nervous (Ner)	Paralysis, memory longevity, dizziness, antidepressant, chito (epilepsy)	12	11	0.090	9	9	0	0.045
Sensory (Sen)	Eye, ear	3	3	0	0	0	0	0

**Fig. 3:**
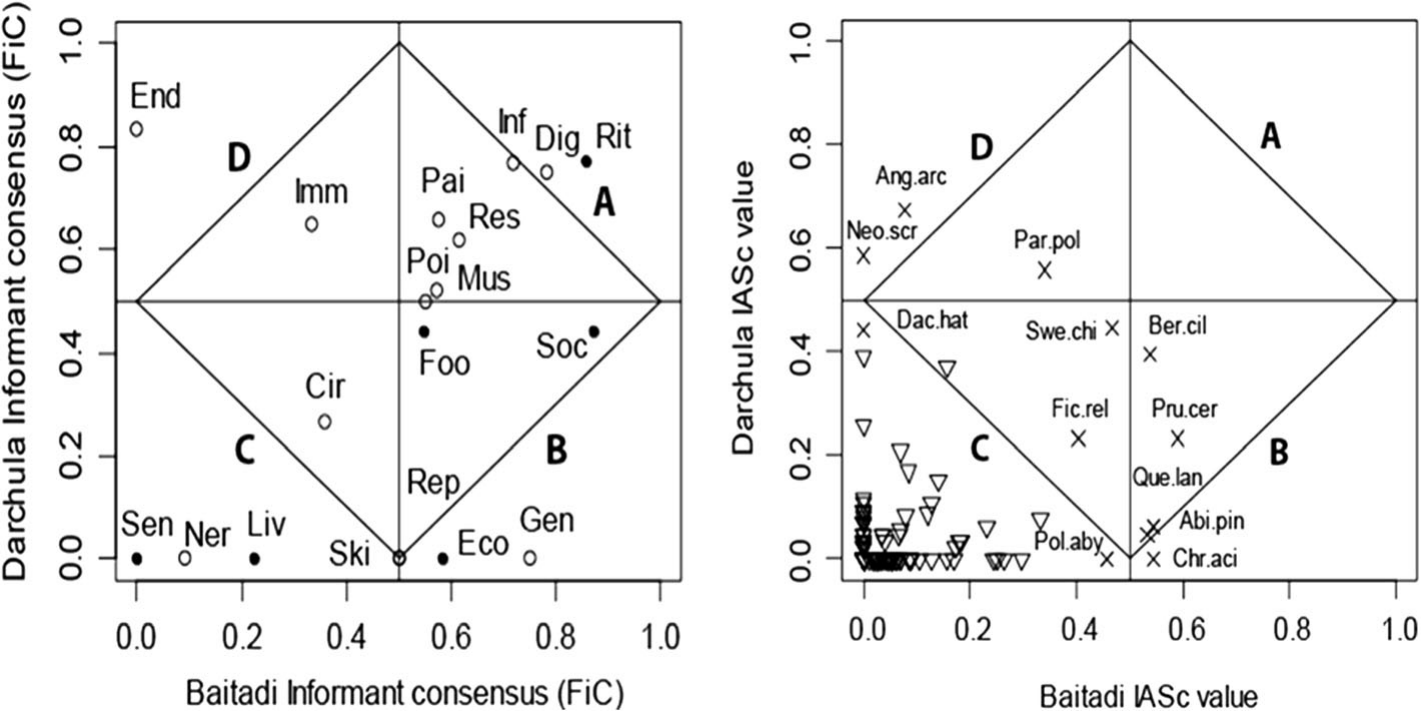
Cultural consensus matrix of two groups (Baitadi and Darchula participants). Right—species: Abi.pin = *A. pindrow*, Ang.arc = *A. archangelica*, Ber.cil = *B. ciliata*, Chr.aci = *C. aciculatus*, Fic.rel = *F. religiosa*, Neo.scr = *N. scrophulariflora*, Par.pol = *P. polyphylla*, Pol.aby = *P. abyssinica*, Pru.cer = *P. cerasoides*, Que.lan = *Q. lanata*, and Swe.chi = *S. chirayita*. Left—use category: Cir = circulatory, Dig = digestive, Eco = economic, End = endocrine; Foo = food, Gen = genito-urinary, Imm = immune, Inf = infections, Liv = livestock, Mus = musculoskeleton, Ner = nervous, Pai = pain, Poi = antipoisoning, Rep = reproductive, Res = respiratory, Rit = ritual, Ski = skin-cutaneous, Sen = sensory, and Soc = social

Of the 19 categories we grouped, 16 categories were found significant for both districts. The categories with less than three use reports were not considered for further analysis (Fig. 3, left). The recorded FiC values for 19 categories were ranged from 0 to 0.87 in both of the districts revealed that the use reports are species-specific and less shared among the participants and district groups. In comparison, highest FiC value was reported for sociocultural and livelihood use (wood, fuel, fodder) (FiC 0.87) in Baitadi District in contrast to the highest FiC value (0.83) recorded for medicinal use treatment of gallstone and endocrine aliments in Darchula District. The second highest FiC 0.85 and 0.81 respectively for Baitadi and Darchula districts were recorded for ritual purpose use (Table 1).

We considered IASc > 0.5 as a cutoff value for identifying the highly consented species. Results showed that there are eight species with IASc value > 0.5 (Table 2). The comparison of informant agreement values between the Baitadi and Darchula districts was significantly different (*p* <  0.001). In order to investigate the relationship between plant knowledge and consensus at the group (cultural) level, plant species were ranked according to their IASc value. In Baitadi, an IASc value > 0.5 was obtained for five species: *Prunus cerasoides*, *A. pindrow*, *Chrysopogon aciculatus* (Retz.) Trin., *Bergenia ciliata* (Haw.) Sternb., and *Quercus lanata* Sm. whereas only three species: *Angelica archangelica*, *N. scrophulariflora*, and *P. polyphylla* were recorded as the highest IASc value > 0.5 species in Darchula (Table 2, Fig. 3). Species with < 0.5 IASc are given in Additional file 1.

**Table 2:** Plant species with high IASc scores > 0.5

Scientific name (abbreviation)	Family	Baitadi				Darchula			
		Use types #	Use reports	Participants (*n*)	IASc	Use types #	Use reports	Participants (*n*)	IASc
*Abies pindrow* (Royle ex D.Don) Royle	Pinaceae	1	31	31	0.543*	2	4	4	0.062
*Angelica archangelica* L.	Apiaceae	4	9	7	0.076	2	30	30	0.673*
*Bergenia ciliata* (Haw.) Sternb.	Saxifragaceae	7	42	36	0.539*	3	20	19	0.395
*Chrysopogon aciculatus* (Retz.) Trin	Poaceae	1	31	31	0.543*	1	1	1	0
*Neopicrorhiza scrophulariflora* (Pennell) D.Y. Hong.	Plantaginaceae	1	1	1	0	4	31	28	0.586*
*Paris polyphylla* Sm.	Melanthiaceae	5	27	23	0.341	4	28	27	0.558*
*Prunus cerasoides* Buch.-Ham. ex D.Don	Rosaceae	4	47	36	0.590*	1	10	10	0.232
*Quercus lanata* Sm.	Fagaceae	7	58	34	0.533*	2	4	3	0.046

*n* = respondents in district, total respondents (*N*) = 100

*Significant

Analysis of use value of plants in two different cultures revealed that three species *P. polyphylla*, *N. scrophulariflora*, and *A. archangelica* are medicinal in uses and emerged in quadrant “D” with their high IASC value (0.55–0.67) in Darchula District (Fig. 3, right). Five species appeared in quadrant “B” (*A. pindrow*, *B. ciliata*, *C. aciculatus*, *P. cerasoides*, and *Q. lanata*) with high IASc value (> 0.5) were important in Baitadi District. Of the five > 0.5 IASc species in Baitadi, only one species *B. ciliata* was used as medicinal, and the rest four were used for sociocultural purposes. *A. pindrow* was valued as a timber/wood species, *Q. lanata* as a fire-wood and fodder, *C. aciculatus* as forage for livestock feeding, and *P. cerasoides* for ritual ceremonies. The species appeared in quadrant “A” were highly consented in both districts whereas those that appeared in quadrant “C” were insignificant in uses and consensus. None of the species was highly consented in both district indicated that the use value of plant species was specific to the district groups and culturally divergent.

### Intracultural knowledge

**Fig. 4:**
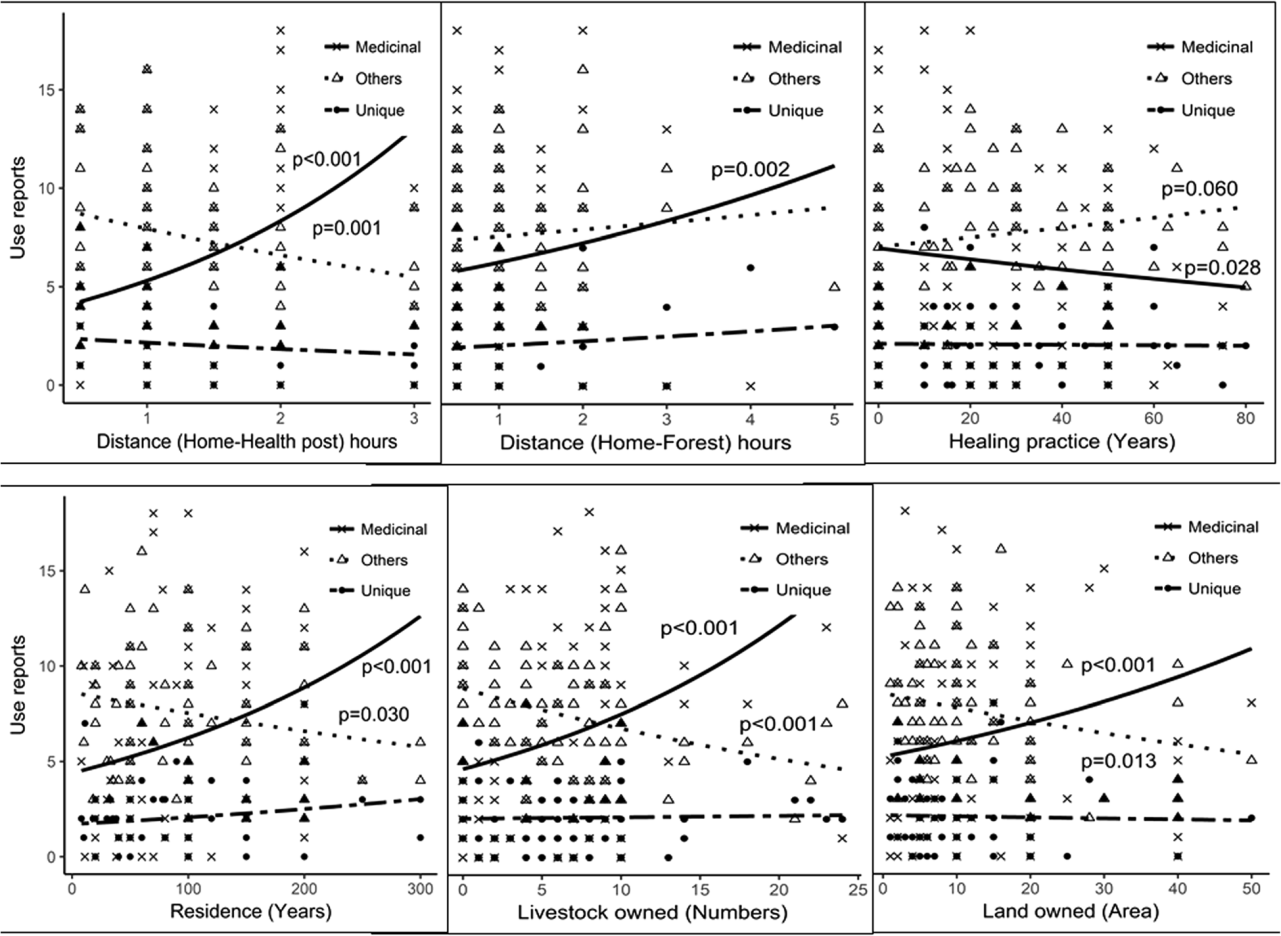
Generalized linear model regression of plant use knowledge of participants along the gradients of sociocultural asset, length of residence, and the time required to access resources. Column represents number of use reports cited by participants

A total of 15 sociocultural factors were tested against the three types of plant use reports: medicinal use reports-MUR, other use reports-OUR, and unique use reports-UUR (Additional file 2). Of the eight continuous variables, four—length of residence, possession of livestock and land, and the distance required to access health post—were found significant (*p* < 0.001–0.03) for both MUR and OUR (Table 3, Fig. 4). Other factors such as distance required for forest access, length of healing practice, and household size of the participants were insignificant for MUR; however, OUR was partially influenced. The unique use report (UUR) was indifferent to continuous variables at all. UUR was also insignificantly different at all categorical variables except the opportunity access and education of the participants (*p* = 0.045 and 0.043, respectively) (Tables 3 and 4).

**Table 3:** Generalized linear model (Poisson’s) regression coefficients of continuous variables against plant use reports

SN	Explanatory variables (continuous)	Medicinal (MUR)	Others (OUR)	Unique (UUR)
1	Length of residence (year)	< 0.001***	0.030*	0.077
2	Livestock owned (number)	< 0.001***	< 0.001***	0.795
3	Land owned (number)	< 0.001***	0.013*	0.698
4	Home-health post-distance (hour)	< 0.001***	0.001***	0.145
5	Home-forest distance (hour)	0.002**	0.356	0.253
6	Household size (number)	0.001**	0.148	0.141
7	Healing practice (year)	0.028*	0.060	0.854
8	Age (year)	0.556	0.115	0.651

> 0.05 non-significant (Ns)

***Highly significant

**Moderately significant

*Low significant

**Table 4:** Analysis of deviance of three types of use reports (MUR, OUR, UUR) in response to livelihood, ethnicity, gender, occupation, education, language spoken, and availability of food in generalized linear model fit with Poisson coefficients

Explanatory variables (categorical)	Factors	MUR		OUR		UUR	
		Mean	*p*	Mean	*p*	Mean	*p*
Livelihood	Baitadi/sedentary (suburban, hill) (57)	6.75	< 0.001***	8.92	< 0.001***	2.06	0.99
	Darchula/semi-nomadic (rural, mountain) (43)	8.69		2.88		2.04	
Access to opportunity	Privileged (Brahmin, Chhetri) (72)	7.40	0.27	7.38	< 0.001***	2.24	0.045*
	Underprivileged (Byanshi, *Dalit*) (28)	8.07		3.60		1.60	
Gender	Male (68)	7.59	0.99	6.72	0.023*	2.26	0.49
	Female (32)	7.58		5.49		2.36	
Occupation	Healers (77)	7.79	0.17	5.83	< 0.001***	1.99	0.79
	Non-healer (23)	6.91		7.99		2.02	
Languages spoken	≤ 2 (81)	7.46	0.36	6.99	< 0.001***	2.26	0.51
	> 2 (19)	8.10		5.72		2.07	
Education	Non-literate (51)	7.78	0.47	6.03	0.23	2.09	0.043*
	Literate (49)	7.38		6.63		1.78	
Food availability	< 6 months (72)	7.51	0.65	6.36	0.84	2.04	0.75
	> 6 months (28)	7.78		6.25		2.14	

***Highly significant, **Moderately significant, *Low significant, > 0.05 Non-significant (Ns)

The participants living in the study area for generations possessed the highest knowledge of plant use, which was significantly different (*p* < 0.001) from participants who moved there more recently. Higher plant use knowledge was also associated with the participants who had a larger number of livestock (*p* < 0.001) and greater land size (*p* < 0.001) and family member (*p* = 0.001); however, the latter was insignificant (*p* = 0.148) to OUR. The settlement: distance from home to a health post (*p* < 0.001) and home to forest (*p* = 0.002) was positively associated to MUR; however, the OUR knowledge was insignificant to the distance of home to the forest. Unlike other studies, age of the participants did not show any statistically significant variation (*p* = 0.55) on means of the knowledge of plant use at all (Table 3). Contrarily, the healing experience of the participants was negatively associated with MUR (*p* = 0.028). Mixed result was obtained when the participants were categorized into healers and non-healers by their occupation and assessed their knowledge of MUR (*p* = 0.17) and OUR (*p* < 0.001). Though healers were more knowledgeable on MUR (7.79), it was statistically insignificant (*p* = 0.17). They also provided less knowledge about OUR (5.83) and UUR (1.99) than the non-healer respondents.

In the study area, the variation of plant use knowledge was closely related to the categorical variables: livelihood, access to opportunity, gender, occupation, and language spoken by the participants, among which livelihood was significant (*p* < 0.001) for both MUR and OUR. The latter four were significant for OUR and UUR. Even though participants responded differently about the plant recognition, identification, collection, and uses [112], this finding was beyond the scope of this present study, and we analyzed only the use responses of the participants. We partially rejected our null hypothesis and found that a significant difference (< 0.001) on plant use knowledge was between the participants with agribusiness and agro-pastoral livelihood and hill/suburban and remote/rural setting.

## Discussion

### Useful plants and their use values

The account of 122 useful plant species was about 50% of the total 255 plant species recorded from the districts. The record of higher number of medicinal plants and edible fruits from herbs and trees respectively was in line with the findings of Toledo et al. [19]. The extensive usage of plants for livelihood and health care indicates that this is clearly an important part of the culture. Poaceae was a richly represented plant family with eight useful plant species followed by Moraceae with seven, Apiaceae with six, and Asteraceae and Fabaceae with five each. Our record of higher number of useful plant species from Poaceae, Asteraceae, and Fabaceae, the most species-rich plant families in the world, was supported by the hypothesis of Moerman [113]; families with abundant species emerges with an abundance of useful species. Asteraceae and Poaceae are the richest plant families in Darchula [50, 114]. Records of higher numbers of useful plant species from the plant families Asteraceae, Fabaceae, and Rosaceae were already manifested in and around KSL, Nepal [15, 49, 74, 77, 99, 115, 116]. Family-wise, the IASc value also revealed that the families Poaceae and Fagaceae were highly consented as useful (IASc 0.79 and 0.59, respectively) followed by Rosaceae and Pinaceae 0.54 each.

### Intercultural analysis

The species with high consensus (> 0.5) were greatly varied at use types and district level. Out of 122 useful botanical taxa documented in this study, only three species *A. archangelica*, *P. polyphylla*, and *N. scrophulariflora* received the highest consensus (IASc > 0.5) among the participants of Darchula. All three species were used as medicine. The use of *N. scrophulariflora* against fever and headache was folkloric with higher IASc in KSL, Nepal, supported by the earlier findings [15, 115, 116]. *P. polyphylla* was considered as a common antidoting plant and *A. angelica* as effective herbal for indigestion in traditional medicine. Species *A. pindrow*, *B. ciliata*, *C. aciculatus*, *P. cerasoides*, and *Q. lanata* received the highest IASc (> 0.5) among the participants of Baitadi. All species except *B. ciliata* were used for livelihood and ritual uses. *P. cerasoides* emerged as important for participants for ritual uses. Other ritual species with less IASc (< 0.5) reported in this study were *Ficus religiosa L.*, *Mangifera indica L.*, *Butea monosperma* (Lam.) Kuntze, and *Selinum wallichianum* (DC.) Raizada & H.O. Saxena*.* All these species have been previously reported as ritual and culturally valuable in KSL, India [40, 115]. *Aegle marmelos* (L.) Correa, *F. religiosa*, *P. cerasoides*, *M. indica*, and *P. emblica* L. are cultural and religious plants of Nepal [117]. Participants considered these five trees as *Panch pallav* (a ritual assortment of five holy leaves) and used them ritually during three major events of life: birth, marriage, and funeral ceremonies. Besides these, *Artemisia indica* Willd. (Kurjo), *Ocimum tenuiflorum* L. (Tulsi), and *Syzygium cumini* (L.) Skeels (Jamun) were also reported as ritual plants in this study.

Other high IASc species such as *A. pindrow*, *C. aciculatus*, and *Q. lanata* from Baitadi District were regarded as useful for livelihood as wood, fuel, and fodder. Since we considered the Baitadi District as relatively more accessible than Darchula, all the useful plants with high consensus of the district are associated with the accessibility and availability. Of the five high IASc (> 0.5) species, *F. religiosa*, *P. cerasoides*, and *Q. lanata* are abundant at nearby settlements. The high consensus value species were not only being frequently used in the study area, they were also reported as highly useful in other villages of study districts [29, 99, 101, 118]. Of the total eight species with high IASc (> 0.5), four were for medicinal purposes and four were for non-medicinal (livelihood and ritual) purposes. This result illustrates that despite living in the same cultural landscape, the species were differently valued, perhaps because of different ethnic groups, accessibility, use values, and livelihood strategies. This result was supported by the fact that none of the highest IASc common species was emerged in quadrat A. Darchula District is relatively undisturbed and more distant than Baitadi District. Traditional healers often cite the distant and undisturbed sites as refuges for both high quality and quantity of medicinal plants and products. Other studies report similar findings from adjoining areas of India [98] and other parts of the world [119, 120], where a higher number of indigenous species with medicinal usage are being used at remote and higher altitudes.

The result of IASc was supported by informant consensus factor FiC. Naturally, both assess the consensus; however, the first assesses the consensus at species level whereas the latter evaluates at use types/category. The highest FiC (0.87) was reported for sociocultural livelihood use (wood, fuel, fodder) followed by 0.85 for cultural/ritual uses in Baitadi District. In contrast, the highest FiC value (0.83) was recorded for medicinal use in the treatment of gallstone and endocrine aliments in Darchula district. Higher FiC values indicate the consent of informants on the specific use of a plant in a traditional use system [121]. The average FiC value of Baitadi and Darchula districts showed that ritual use of plants possesses the highest FiC value (0.81) followed by digestive system disorder (0.76) and infections (0.74). Higher consensus on ritual uses was consistent with the sacredness of the area. Baitadi and Darchula districts are the southern parts of the KSL, and they are well known for culture- and religious-based taboos, religious fencing, and a high number of sacred peaks [57, 58, 73, 80].

The frequent use of medicinal plants to cure ailments could be attributed to the high preponderance of digestive and infectious disorders in the area. This account aligns with the government report [70, 122] stating the prevalence of diarrhea and dysentery in far-western Nepal. The health and development index, partly a measure of nutritional status, ranks the study districts (Baitadi and Darchula) 66 and 62 among 75 districts of the country [70]. Health was further jeopardized by food deficiencies [39, 47, 89]. The situation was provoked by people drinking contaminated water, eating improperly stored and spoiled foods, and conditions of poor nutrition. The highest number of taxa and use reports for treatment of stomach disorders in Darchula District was also observed by Aryal et al. [29]. Since our study area is a food deficit [123] and 72% participants had food deficiency > 6 months year^−1^, the tradition of wild plant collection, use, and management was a common strategy to combat poverty paired with geo-ecological constraints and sacredness of the landscape, resulting in insignificant difference between the groups of people with food deficiencies. The role of plant collection in Darchula and Baitadi districts in complementing food availability was appreciated [29]. The low consensus of plant use between Baitadi and Darchula districts could be due to both cultural divergence, varied accessibility, and physiographic heterogeneity.

We found a cultural distinction in collection, use, and conservation of plants. Mountainous pastoral communities of Darchula people often collect plants from remote areas for medical ethnobotany. They reported that their household economy was complemented by 8.13 ± 4.75% from the sales of medicinal plants of rural undisturbed areas whereas less (5.96 ± 5.46%) was reported in Baitadi District. The sedentary hilly farmers of Baitadi District value plants more for social, ritual, and livelihood, and often generalist collectors from nearby areas of settlement forage there. Conversely, the forage from distant forest areas in Darchula was associated with quality products, traditional medicine, and elder healers. Since the Darchula people are occupational traders of medicinal plants, they have long been foraging medicinal plant products from remote and relatively undisturbed areas and selling them to lowland groups and to India and China for food grains. While collecting medicinal plants, many collectors worship and pray to plants and acknowledge the spiritual powers for quality products [91] because they believe that plants become more medicinal when processed both spiritually and materially [65, 66]. Thus, mountains are valued as sacred sites and destinations for livestock grazing and the collection of quality medicinal plants for rural household [124, 125].

People often trade medicinal plant products such as *Allium wallichii* Kunth (Ban lasun), *A. hypsistum* (Jimbu), *B. ciliata* (Vedaite), *Delphinium denudatum* Wall. ex Hook.f. (Nirmasi), *P. polyphylla* (Satuwa), *Nardostachys grandiflora* DC. (Jatamansi), *N. scrophulariflora* (Katuko), *O. sinensis* (Yartsagumbu), *Zanthoxylum armatum* DC. (Timur), etc. The pursuit of collection, bartering, and trade of medicinal plants was dated back to the 1960s [8, 10, 126]. The plants were bartered for grains in the districts before trading [69], and the tradition was balanced until the 1970s [27, 73, 84]. When the district forest offices were set up in the 1970s, the institutionalized trading practice was started, and abundant species such as *B. ciliata*, *S. chirayita*, and *Valeriana jatamansii* Jones were collected for trading. Nepal Government records (2000–2016) show that *S. chirayita* was traded 3.6 tons in 1999, 4 tons in 2008, and 2.7 tons in 2016 from both districts [127]. Other local medicinal plants *Asparagus racemosus* Willd., *Berberis asiatica* Roxb. ex. DC., and *Cinnamomum tamala* (Buch.-Ham) Nees & Eberm. were also collected from both districts in 1999 for marketing purpose [127]. The households involved in collection of *O. sinensis* from remote alpine pasture in Darchula District gathered 140 kg in 1998 to 1440 kg in 2004 [128], 4500 kg in 2008 [129], and 5000 kg in 2016 [126] indicating the steady growth in collection of high value medicinal plants and products in an effort to earn quick economic returns. Although an in-depth investigation of trade dynamics in the districts was outside of the scope of this study, it is noteworthy that several informants recalled the bartering, citing the importance of traditional trade and lamenting the declining medicinal plant species because of fluctuating and market-driven collection.

Such directed and culture mediated collection and use of plants for personal gain (health and income) over communal incentives (social and ritual significance) yield a direct impact on resource conservation. Persistence of intercultural divergences occurred in specialized medicinal and ritual uses of plants or trade of certain species. The distinction of knowledge of cultural groups substantiates the hypothesis that cultural differences play an important role in the transfer and maintenance of indigenous knowledge. Despite the persistence of different strategies for collecting and utilizing plants, the geographic adversities have strengthened a homogenous strategy of employing locally available plants for livelihood and culture. The way of life in rural hills/mountains involves adjusting to the difficult environment, food deficits, and limited accessibility, with appropriate strategies [94]. Thus, the tie between people, plants, and places is strong and inseparable in Darchula and Biatadi districts of KSL, Nepal.

### Intracultural analysis

An intracultural difference of plant use knowledge at the individual level was evident with the cultural heritage. Participants’ years of residence in the study area, the size of their livestock herd and land, family size, and the time required to access a health post (*p* ≤ 0.001) were positively associated with the ethnomedicinal knowledge of plant use. The greater length of residence in an area helps in accumulating greater vast knowledge required to use the resources wisely [130]. There were two household lineages in Baitadi District, living there for more than 300 years, that described the usefulness of 16 plants and 20 use reports each, higher than the average record (use reports, 14.34; useful plants, 12.68 person^−1^). This supports the tenet of a positive association between longer residence and greater knowledge.

Limited access to health centers compels people to use local resources for their primary health care, inferring the role of geography. *N. scrophulariflora*, *A. archangelica*, and *P. polyphylla* are abundant in remote and wild forests and highly medicinal (IASc > 0.5). *O. sinensis* that grows well in alpine pastures is folkloric as a tonic and antipyretic in Darchula. This could be one reason that local communities forage the wild and distance sites for quality products regardless of the distance and geo-ecological constraints. While herding, summer grazing, and ascending for the collection of medicinal plants, local people share knowledge of plant identification, collection, uses, and management. Horizontal transmission of knowledge among shepherds and herders while herding and transhumance contributes to a high level of knowledge sharing, thus aiding conservation knowledge. Cultural values often aid the knowledge of plant use and collection in high altitude areas [5]. The transhumance practice generates a deep connection to the environment, enhanced by the fact that during this time, the families have limited choices [131]. In our study area, the indigenous livelihood strategies such as animal husbandry, transhumance with their livestock, and medicinal plant collection were interlinked. Livestock ownership associates positively with ethnobotanical knowledge in mountains [132]. The sizes of livestock herd, land owned, and family member were slightly correlated (household~land 0.38, household~livestock 0.41, and land~livestock 0.48). Larger families own larger herds [95, 133] which is important for the transhumance community [134]. Household size increases brought about an increased utilization of medicinal plants, perhaps due to economic pressure in larger families. The positive correlation between medicinal plant use and age has been noted elsewhere [117, 135]. However, we did not obtain a significant relationship between the participants’ age and the knowledge of plant use and accepted the null hypothesis. No difference of plant use knowledge along the age was also reported by McMillen [136].

OUR and UUR were insignificant along age whereas the MUR was slightly significant indicating that the knowledge of medicinal plant use insignificantly decreased as age increases. Our results indicated that plant use knowledge was less varied among the participants because in situ transmission is underway when needed as described by Phillips and Gentry [3] and Paniagua-Zambrana et al. [7]. Age group (40–59, 60–79, > 80 years) analysis also did not reveal significantly different MUR knowledge (*p* = 0.46–0.95) among them; however, the age group 40–59 years possessed the higher MUR 8.19 ± 0.53 comparable to MUR 7.2 ± 0.79 of the > 80 years age group. A non-existent relation (*p* = 0.32) was already reported from adjoining villages of our study sites [75] and semi-arid and transhumance communities of Patagonia, Argentina (*p* = 0.34) [137]. Earlier studies argue that the age group 65–75 holds a greater knowledge of plant use [138]. Guimbo et al. [139] found that gender and age have strong effects on the local knowledge of useful plants. However, in this study, the role of both factors was insignificant in differentiating the knowledge of plant uses. Neither MUR nor UUR was significantly different in relation to gender. However, the OUR was varied (*p* = 0.023) and was higher (6.72 ± 5.2) among the male participants. This result was supported by the gendered division of labor where males are often engaged in summer grazing, livestock herding, and extraction of plants from inaccessible sites that would take them to relatively remote and distant sites. Pfeiffer and Butz [140] reported that plant use is differentiated with men and women due to resource access and their social roles. Women are more likely involved in managing local resources that are available nearby [141] and are associated with anthropogenic landscapes [34].

A weak association of age and plant use knowledge could be attributed to the (non-random) selective samples and the unwillingness or inability to share information explicitly by the elderly groups. The secrecy of medicinal plant knowledge is a common practice in different parts of the world [142], and it was common among the traditional healers in our study area. In the *Baidhya* tradition—a local healing tradition in far-western Nepal [13, 75, 91]—the main knowledge of healing is kept with sanctity and secrecy and is confined to few healers. Healers generally believed that the medicines would lose their efficacy if too many people knew about their use. Elders generally consider Sundays and Wednesdays as good days in collection of medicinal plants while Saturdays, Mondays, and Tuesdays are often avoided due to religious considerations. Thus, the plant knowledge often belongs to the specialty domain of a culture and limited number of individuals and may be secretive for this purpose [106], whereas other general knowledge is widely available to other community members and freely shared. Species-use curve showed that the curve attained asymptote in Baitadi District (range of respondents age 40–96 years). However, the curve was not leveled-off in Darchula District (range of respondents age 41–102 years). As sampling effort increases in Darchula, more elders/healers will be encountered, and more species are likely to be recorded. Thus, if we consider elders as sample respondents for ethnobotanical studies, a greater number of samples are required in order to find the saturation point of the community consensus and statistically significant values of knowledge [143].

General knowledge such as OUR was commonly shared among communities through cultural learning (*p* = 0.06) whereas the specialty knowledge like use of medicinal plants for ailments (MUR, − 0.028) and unique use reports (UUR, 0.85) were transmitted through closed and vertical sharing with directed and dedicated apprenticeships under the tutelage of senior practitioners, resulting in constrained transfer. There were 75% (57 out of 77) traditional healers that learned medicinal plant use knowledge from their parents and grandparents (vertical transfer), whereas 25% healers learned themselves or from peer healers (horizontal transfer). Vertical knowledge transmission is often associated with family members and the sharing of secretive knowledge [13, 55]. Even though the difference was insignificant, the knowledge of healers (MUR 7.79 ± 0.34) influenced the use of medicinal plant collection and use, comparable to that of non-healers (MUR 6.91 ± 0.56). Moreover, the knowledge was effaced by the decline of traditional healers and their limited sharing [29, 75]. Lower population growth in the districts (0.70–0.92) than the national average (1.44) and higher than national outmigration rates (absentee population of 7.51% higher than the national average 7.23%) [70] produced a decline in the number of healers and those with traditional knowledge. Families from the region have migrated to cities and lowlands, resulting in an accentuated decline in indigenous land use and plant collection and use. There were about 15 traditional healers in each study village in KSL, Nepal in 2014 whereas only 6 in each village were reported in the present study. The number of healers is decreasing fast (about 7% per annum) in KSL, Nepal [75], resulting in threatened knowledge of plant use.

The participants who are non-literate had also significant UUR (*p* = 0.043). MUR was also influenced by the literacy level of participants (non-literate 7.78 ± 2.81, literate 7.38 ± 3.14). Higher knowledge of MUR among the non-literate participants could be attributed to their direct association with forest and natural resources and frequent and first choice of traditional and home-based medicines for ailments. Thorsen and Pouliot [144] showed that the traditional medicine is the first choice and ultimate hope of recovery of chronic illness among rural elders and non-literate people of Nepal. The prevalence of traditional medicine was attributed by the limited number of health workers (one health worker for every 3300 people in Baitadi and 1900 in Darchula) [121] than the traditional healers (one for every 100 people) in Nepal [145, 146] and the belief and long-rooted tradition/history of using quality medicinal plants in rural and remote areas. A similar account of higher traditional knowledge of plant use among non-literate participants was reported by Umair et al. [147] in the Pakistan Himalaya. The participants who speak only one or two dialects were significantly knowledgeable to OUR (*p* < 0.001).

Among three response variables, MUR and OUR were significantly different (*p* < 0.001) between the participants of different livelihood type: semi-nomadic communities from rural mountains of Darchula (MUR 8.69 ± 3.01) and sedentary communities with the suburban setting of Baitadi (MUR 6.75 ± 2.67). Most of the people from Darchula District inhabiting in remote areas are occupational medicinal plant collectors and traders and do summer grazing, travelling during transhumance, and often seek high-value medicinal plants for trading purposes. The underprivileged groups (Byanshi and *Dalit*) were knowledgeable on MUR (8.07 ± 3.2) however insignificant (*p* = 0.27). They were less knowledgeable on general use reports (3.60 ± 3.14, *p* < 0.001) and unique use report 1.60 ± 1.37, *p* = 0.045). The *Dalit* are disadvantaged groups of the country and have limited access to the natural resources in Darchula District because of the sociocultural and caste system [72, 88, 148, 149]. Their limited access could have limited their plant use. MUR was specific to indigenous underprivileged minority groups and rural agro-pastoral livelihood type because of the subsistence economy and historical connectivity to the medicinal plants [54]. OUR was folkloric to their counterpart with the highest significance level (*p* < 0.001). OUR was significantly different (*p* < 0.001–0.023) for five out of seven categorical variables: access to opportunity, gender, occupation, language spoken, and livelihood type. The higher mean values of OUR 7.38 and 8.92 compared to 3.60 and 2.88 respectively of privileged groups and from sedentary communities of Baitadi District indicated that the communities living with more amenities and better privilege were more knowledgeable about general non-medicinal uses (OUR) such as uses for livelihood and rituals and divergent from Darchula people.

Sedentary communities living in low-elevation environments have accommodated livelihood to an economic system based on agriculture, markets, and jobs. Being close to markets, availability of medical supplements in markets and pursuance of agribusiness livelihood contribute to the reduced dependency on medicinal plant resources and contribute less to MUR in Baitadi lowlands. The use of modern medicine, increasing road linkages, decreasing plant resource availability, and agricultural intensification are responsible for the changing medicinal plant use knowledge in Baitadi [35]. The use knowledge of ritual and religious plants is still persistent regardless of the modern facility if cultural supplements are unavailable in the markets. High culturally shared species (IASc) are found nearby settlements and common for general uses whereas the specialty use (such as MUR) species are foraged by trained personnel, guided methods and from the remote undisturbed sites [118]. The difference in plant use knowledge may help in diversifying the livelihood strategies in accordance with the environment [150].

## Conclusions

The extensive usage of plants for socioeconomy, livelihood and rituals indicate that the plants, people, and culture in the Nepal Himalaya are inseparable. We found that the knowledge of plant use seems to follow a pattern according to available useful plants as well as the cultural significance of the landscape. However, the latter prevails. The use knowledge of plants coincided with the richness of species and plant families. Foraging by the agro-pastoral communities from the remote undisturbed areas for quality products and medicines in Darchula District was divergent with the collections from ruderal and nearby areas in Baitadi District by generalist collectors for ritual uses. General knowledge of plant use such as OUR was commonly shared among communities through cultural learning whereas the specialty knowledge like the use of medicinal plants for ailments (MUR) and unique use reports different from peers (UUR) was transmitted through closed and vertical sharing with directed and dedicated apprenticeships under the tutelage of senior practitioners. The regional particularities—geographic (isolation, accessibility, settlement) and cultural (livestock, land, length of residence and livelihood, and rites)—seem relevant in explaining the differences in plant use knowledge in Baitadi and Darchula districts, Nepal. These results contribute to a growing body of literature that expands our understanding of patterns of knowledge of useful plants across culture and geography.

## Additional files

Additional file 1: Species use reports and IASc at district level. (XLSX 31 kb)

Additional file 2: Socioeconomic variables and their analyses. (CSV 13 kb)

## Abbreviations

FiC: Informant consensus factor
GoN: Government of Nepal
IASc: Index of agreement on species consensus
KSL: Kailash Sacred Landscape
MUR: Medicinal use report
OUR: Other use report
UUR: Unique use report
